# SHOC2 plays an oncogenic or tumor-suppressive role by differentially targeting the MAPK and mTORC1 signals in liver cancer

**DOI:** 10.1093/lifemedi/lnae023

**Published:** 2024-05-23

**Authors:** Xiahong You, Longyu Dou, Mingjia Tan, Xiufang Xiong, Yi Sun

**Affiliations:** Cancer Institute (Key Laboratory of Cancer Prevention and Intervention, China National Ministry of Education) of the Second Affiliated Hospital, and Institute of Translational Medicine, Zhejiang University School of Medicine, Hangzhou 310029, China; Cancer Center of Zhejiang University, Hangzhou 310029, China; Cancer Institute (Key Laboratory of Cancer Prevention and Intervention, China National Ministry of Education) of the Second Affiliated Hospital, and Institute of Translational Medicine, Zhejiang University School of Medicine, Hangzhou 310029, China; Cancer Center of Zhejiang University, Hangzhou 310029, China; Department of Radiation Oncology, University of Michigan, Ann Arbor MI 48109, United States; Cancer Institute (Key Laboratory of Cancer Prevention and Intervention, China National Ministry of Education) of the Second Affiliated Hospital, and Institute of Translational Medicine, Zhejiang University School of Medicine, Hangzhou 310029, China; Cancer Center of Zhejiang University, Hangzhou 310029, China; Cancer Institute (Key Laboratory of Cancer Prevention and Intervention, China National Ministry of Education) of the Second Affiliated Hospital, and Institute of Translational Medicine, Zhejiang University School of Medicine, Hangzhou 310029, China; Cancer Center of Zhejiang University, Hangzhou 310029, China; Zhejiang Provincial Clinical Research Center for CANCER, Hangzhou 310029, China; Research Center for Life Science and Human Health, Binjiang Institute of Zhejiang University, Hangzhou 310053, China

**Keywords:** SHOC2, RAS, mTORC1, PTEN, liver cancer

## Abstract

SHOC2 is a scaffold protein that activates the RAS-MAPK signal. Our recent study showed that SHOC2 is also a negative regulator of the mTORC1 signal in lung cancer cells. Whether and how SHOC2 differentially regulates the RAS-MAPK vs. the mTORC1 signals in liver cancer remains unknown. Here, we showed that *SHOC2* is overexpressed in human liver cancer tissues, and SHOC2 overexpression promotes the growth and survival of liver cancer cells via activation of the RAS-MAPK signal, although the mTORC1 signal is inactivated. *SHOC2* knockdown suppresses the growth of liver cancer cells mainly through inactivating the RAS-MAPK signal. Thus, in the cell culture models, SHOC2 regulation of growth is dependent of the RAS-MAPK but not the mTORC1 signal. Interestingly, in a mouse liver cancer model induced by diethylnitrosamine (DEN)-high-fat diet (HFD), hepatocyte-specific *Shoc2* deletion inactivates the Ras-Mapk signal but has no effect in liver tumorigenesis. However, in the *Pten* loss-induced liver cancer model, *Shoc2* deletion further activates mTorc1 without affecting the Ras-Mapk signal and promotes liver tumorigenesis. Collectively, it appears that SHOC2 could act as either an oncogene (via activating the MAPK signal) or a tumor suppressor (via inactivating the mTORC1 signal) in the manner dependent of the dominancy of the MAPK vs. mTORC1 signals.

## Introduction

SHOC2, also known as Sur-8 and Soc-2, was first identified in *Caenorhabditis elegans* (*C. elegans)* as a scaffold protein for RAS and RAF that positively regulated the RAS-RAF-MAPK signal [1, 2]. The SHOC2 is evolutionarily conserved and contains two main domains: an unstructured N-terminal domain and a lengthy sequence of twenty-one leucine-rich repeats (LRRs) at its core [3]. The N-terminal domain binds to RAS and RAF and forms a complex with the two proteins, thereby specifically enhancing the activation of extracellular stimulus-regulated kinase 1 and 2 (ERK1/2) [4, 5]. Additionally, the LRRs of SHOC2 have been shown to interact with various other proteins, including the catalytic subunits of protein phosphatase 1 (PP1c) and the HECT-domain E3 ubiquitin ligase HUWE1 [6–8].

Recently, our group reported that the FBXW7-SHOC2-Raptor axis controls the cross-talks between the RAS-ERK and mTORC1 signals [8]. SHOC2 was identified as a substrate of SCF^FBXW7^ E3 ubiquitin ligase [8]. Upon stimulation by growth factors, SHOC2 is phosphorylated at Thr^507^ by the MAPK cascade, which facilitates its interaction with FBXW7 for subsequent ubiquitylation and degradation. Moreover, SHOC2 acts as a negative regulator of mTOR signal by competing with mTOR for Raptor binding, thus depleting Raptor from the mTORC1 complex inactivates mTORC1 [8, 9].

Cordeddu et al. [10] initially reported that *Shoc2* mutations, which promote the N-myristoylation of SHOC2 protein and causes a developmental disease, known as the Noonan-like syndrome with loose anagen hair. Furthermore, previous *in vitro* studies have revealed a strong correlation between SHOC2 expression and the proliferation of cancer cells [11, 12]. In fact, SHOC2 enhances the ERK1/2 signals in various malignant cell types [6, 13–18]. Recent studies have also suggested that SHOC2 may hold prognostic value for patients with breast, thyroid, and lung cancers [8, 14, 16, 19]. In line with these oncogenic roles of SHOC2 in human cancers, we recently showed that *Shoc2* deletion in the *Kras*^G12D^ pancreatic tumor model significantly inhibited pancreas growth and progression of murine pancreatic intraepithelial neoplasms (mPanINs) [20]. However, most prior investigations have primarily focused on the canonical role of SHOC2 in regulation of the RAS-MAPK signal, particularly in cancer cells carrying *K-Ras*, *N-Ras*, and *B-Raf* oncogenic mutations or in *Kras*^*G12D*^-induced mice tumor models. Thus far, no *in vivo* study has been reported to elucidate the role of SHOC2 in regulation of the mTORC1 signals.

Hepatocellular carcinoma (HCC) is one of the most common malignant tumors worldwide, leading to high mortality [21]. Despite considerable progress has been made in the elucidation of its etiology and underlying mechanism of disease development, HCC remains a fatal and treatment-refractory disease. Significantly, the mTOR pathway is aberrantly upregulated in approximately 50% of HCC tumors, as determined through a substantial cohort of human HCC tissue samples [22, 23]. Furthermore, a substantial portion of HCC tissues showed down-regulation of the tumor suppressor PTEN (phosphatase and tensin homolog) protein [24–27]. Horie et al. reported that hepatocyte-specific *Pten* deficiency *in vivo* results in steatohepatitis and hepatocellular carcinomas [28]. However, the role of SHOC2 in the liver tumorigenesis, especially in liver cancer model induced by *Pten* loss remains elusive.

In this study, we characterized the role of SHOC2 in liver cancer cells and liver tumorigenesis using both *in vitro* cell culture and *in vivo* mouse models, respectively. In *in-vitro* culture models of liver cancer cells with gain- or loss-of-function approaches, it appears that SHOC2 regulation of growth and survival is dependent of the signal of the RAS-MAPK, but not of the mTORC1 signal. Within two *in-vivo* liver cancer models, we found that in DEN-HFD (diethylnitrosamine plus a high-fat diet) model, hepatocyte-specific *Shoc2* deletion deactivates the Ras-Mapk signal without affecting the mTorc1 signals but has no effect in the progression of liver tumorigenesis. In Pten-null liver cancer model in which the mTorc signal is activated, however, *Shoc2* deletion further activates mTorc1 without affecting the Ras-Mapk signal, leading to accelerated liver tumorigenesis. Thus, it appears that SHOC2 acts as an oncogene by activating the RAS/MAPK or a tumor suppressor by inactivating the mTORC1 signals, respectively, to regulate the growth and survival, and liver tumorigenesis.

## Results

### SHOC2 is overexpressed in human LIHC tissues and positively regulates growth and survival of liver cancer cells

**Figure 1.**
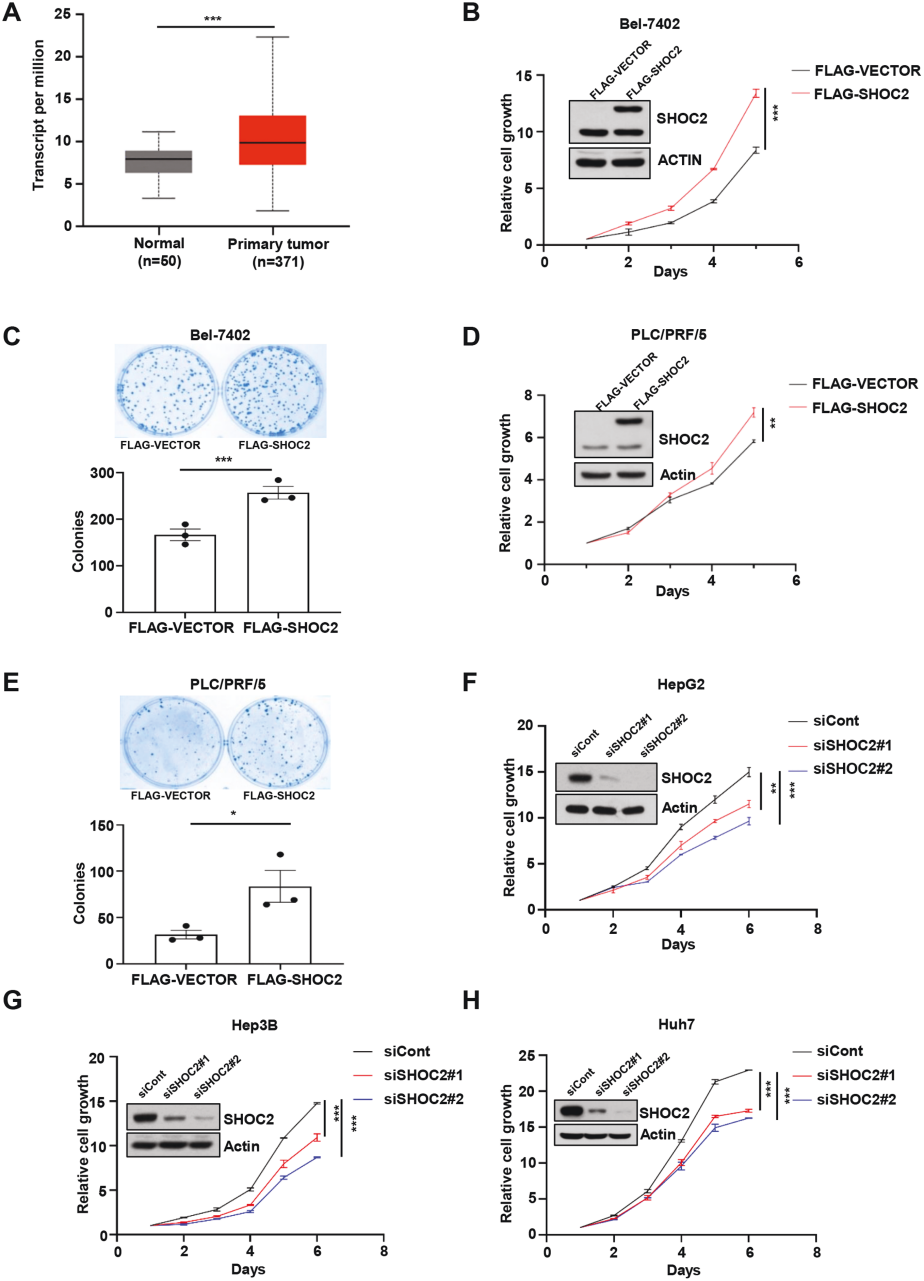
***SHOC2* is overexpressed in LIHC, and positively regulates growth and survival of liver cancer cells.** (A) Differential expression of *SHOC2* between LIHC and normal liver tissues, Data from TCGA database. (B–E) Bel-7402 (B, C) and PLC/PRF/5 (D, E) cells were transfected with the plasmid of FLAG-SHOC2 or FLAG-VECTOR for 24 h; then cells were seeded in 96-well plates in triplicate and subjected to a CCK-8 cell proliferation assay or immunoblotting (IB) (inset), and clonogenic survival assay. The mean ± SEM (*n* = 3) are shown from three independent experiments. (F–H) HepG2 (F), Hep3B (G), and Huh7 (H) cells were transfected with siRNA targeting *SHOC2* or control siRNA (siCont) for 24 h. Cells were then seeded in 96-well plates in triplicate and subjected to a CCK-8 cell proliferation assay or IB (inset). LIHC: liver hepatocellular carcinoma; *, *P* < 0.05; **, *P* < 0.005; ***, *P* < 0.001.

To investigate the possible role of SHOC2 in human liver cancer, we first searched the Cancer Genome Atlas (TCGA) database, and systematically analyzed the mRNA expression of *SHOC2* between hepatocellular carcinoma (LIHC) and normal liver tissues, and found that *SHOC2* is significantly overexpressed in cancer tissues (Fig. 1A). We further detected the SHOC2 levels, along with the basal status of the MAPK and mTORC signals, in nine human liver cancer cell lines and one normal liver cells LO2, and found that SHOC2 levels were relatively lower in Bel-7402 and RLC/PRF/5 cells, but relatively higher in HepG2, Hep3B and Huh7 cells (Fig. S1A). Using a gain-of-function approach in SHOC2 low-expressing Bel-7402 and RLC/PRF/5 cells, we first determined the effect of SHOC2 in controlling the growth and survival. Indeed, ectopic SHOC2 expression at the level comparable to endogenous level promoted the growth and clonal survival of both liver cancer cells (Fig. 1B–E). We then used a loss-of-function approach in SHOC2 high-expressing HepG2, Hep3B, and Huh7 cells and found that *SHOC2* knockdown significantly inhibited the growth of these liver cancer cell lines and clonal survival of Huh7 cells (Fig. 1F–H and Fig. S1B). Since neither HepG2 nor Hep3B cell line formed colonies in culture dishes, we were unable to evaluate the effect on clonal survival upon *SHOC2* knockdown in these two lines. Collectively, SHOC2 is a positive regulator of growth and survival of liver cancer cells.

### SHOC2 positively regulates MAPK but negatively regulates mTORC1 signals

**Figure 2.**
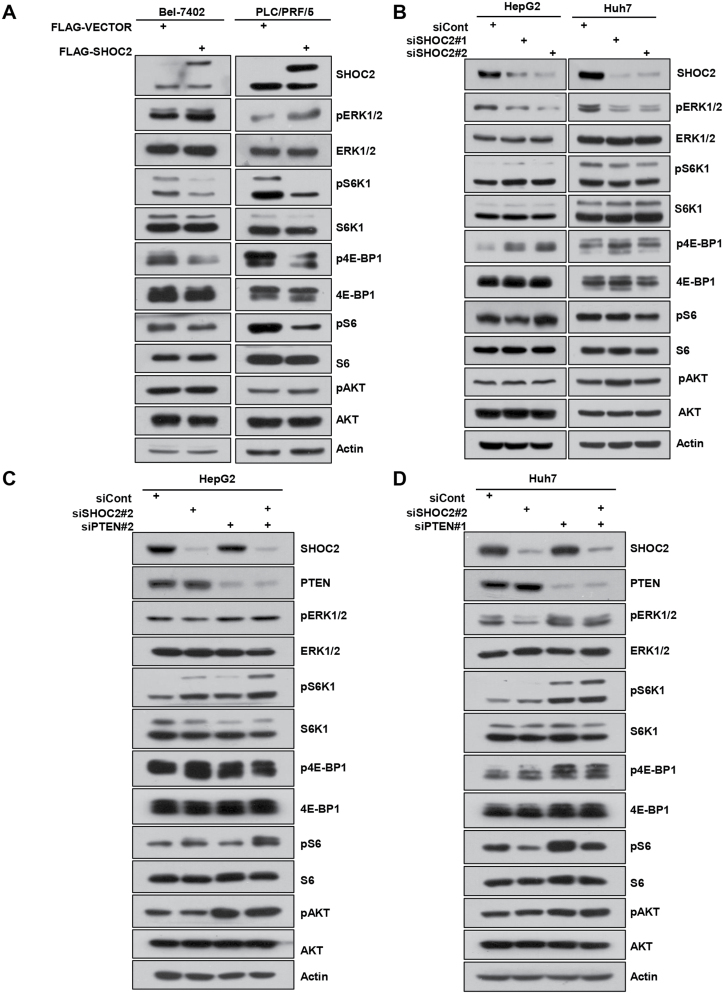
**SHOC2 positively regulates MAPK, but negatively regulates mTORC1 signals.** (A) Bel-7402 and PLC/PRF/5 cells were transfected with plasmid of FLAG-SHOC2 or FLAG-VECTOR for 48 h, followed by IB with indicated antibodies. (B) HepG2 and Huh7 cells were transfected with siRNA targeting *SHOC2* or siCont for 48 h, followed by IB with indicated antibodies. (C, D) HepG2 (C) and Huh7 (D) cells were transfected with indicated siRNAs for 48 h, followed by IB with indicated antibodies.

Our recent study showed that in lung cancer cells, SHOC2 positively regulated the MAPK but negatively regulated the mTORC1 signals [8]. Likewise, we found that ectopic expression of SHOC2 activated the MAPK signal with increased levels of pERK1/2, and at the same time, inactivated the mTORC1 signals with reduction of pS6K1/pS6 and p4E-BP1 levels, but not affecting the mTORC2 signals with unchanged pAKT levels in both Bel-7402 and RLC/PRF/5 cells (Fig. 2A).

Consistently, *SHOC2* knockdown caused significant inactivation of MAPK signals with remarkable reduction of pERK1/2 levels in all three lines of HepG2, Hep3B and Huh7 cells, and at the same time, significant activation of mTORC1 signals with remarkable increase of pS6K1/pS6 and p4E-BP1 levels in HepG2 and Hep3B cells, but not in Huh7 cells (Fig. 2B and Fig. S2A), indicating a cell line dependent effect on the mTORC1 signals. Again, *SHOC2* knockdown had no effect on mTORC2 signal since it did not affect the pAKT levels (Fig. 2B). It is worth noting that the liver cancer cell lines we used all express PTEN (Fig. S1A). Given the fact that SHOC2 manipulations positively regulated the MAPK signals as well as cell growth, while negatively regulating the mTORC1 signals, we concluded that in *in vitro* cell culture models, SHOC2 mainly acts as an activator of the MAPK signals to promote the growth of liver cancer cells in the presence of PTEN.

PTEN acts as an essential negative regulator of the PI3K/AKT/mTOR signaling pathway to suppress cell growth and survival, whereas loss of PTEN, frequently seen in human cancers, results in hyperactivation of the PI3K/AKT/mTOR pathway to promote growth and survival of cancer cells [29]. To determine the effect of PTEN in SHOC2 regulation of the MAPK and mTORC1 signals, we performed siRNA-based *PTEN* knockdown and found a significant activation of the mTORC1 signals, as evidenced by remarkably increased levels of pS6K1, pS6, and p4E-BP1, with minor activation of the mTORC2/pAKT and the MAPK/pERK signals in HepG2 and Huh7 cells (Fig. S2B–C). The double knockdown of *PTEN* and *SHOC2* did cause a greater activation of the mTORC1 signals but without much effect on the MAPK/pERK/pAKT signals particularly in HepG2 cells (Fig. 2C) with a minor increase of the mTORC1 signal in Huh7 cells (Fig. 2D). Collectively, in the *in vitro* cell culture models, SHOC2 mainly negatively regulates the mTORC1 signals of liver cancer cells in the absence of PTEN.

We further determined the effect of *PTEN* knockdown alone or in combination with *SHOC2* knockdown in HepG2 and Huh7 cells, while *PTEN* knockdown promoted, and *SHOC2* knockdown inhibited, respectively, the growth of HepG2 and Huh7 cells (Fig. S3A and S3B) and clonal survival of Huh7 cells (Fig. S3C), the double knockdown of *PTEN* and *SHOC2* canceled the effect of each other, showing minimal, if any, effect on cell growth and clonal survival (Fig. S3A–C).

### SHOC2 regulation of the MAPK and mTORC1 signals under stress conditions

**Figure 3.**
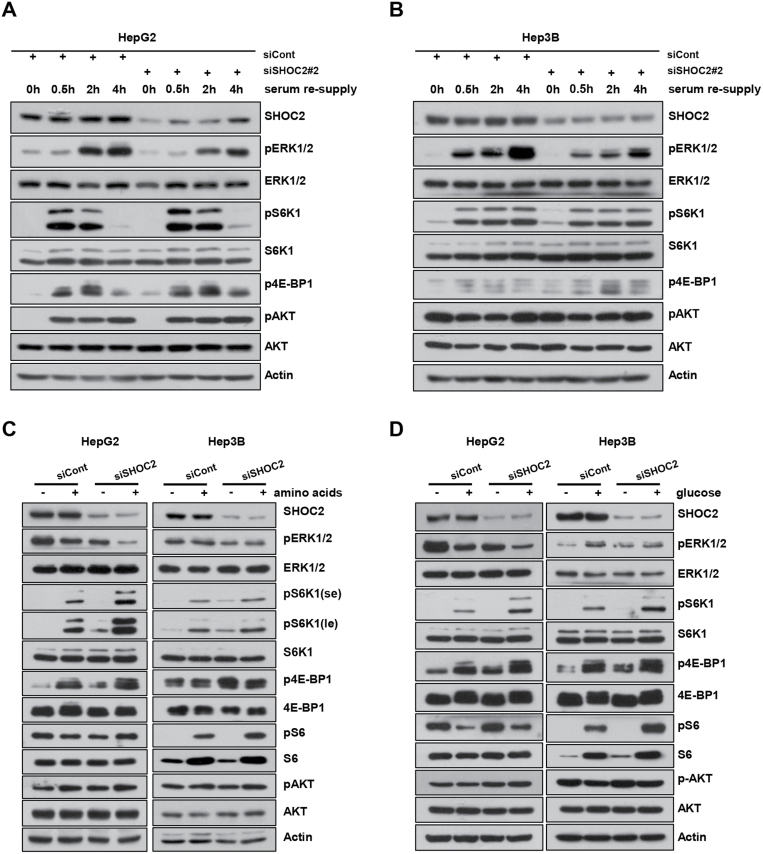
**SHOC2 regulation of the MAPK and mTORC1 signals under stress conditions.** (A, B) HepG2 (A) and Hep3B (B) cells were transfected with siRNA targeting *SHOC2* or siCont for 24 h, followed by serum starvation for 24 h, then serum re-supply for the indicated time periods and harvested for IB. (C, D) HepG2 and Hep3B cells were transfected with siRNA targeting *SHOC2* or siCont for 48 h, followed by amino acids (C) and glucose starvation (D) respectively for 3 h, then re-supply for 20 min and harvested for IB. se: short exposure; le: long exposure.

We next determined the SHOC2 regulation of these two major signals in response to stresses. It is known that both MAPK and mTORC1 signals are inactivated upon serum withdrawal but reactivated upon serum re-supply. To this end, we knocked down *SHOC2* in HepG2 and Hep3B cells, followed by serum starvation for 24 h, then serum re-supply for different time periods. While serum starvation or serum resupply had no effect on SHOC2 levels, serum starvation inactivated both MAPK and mTORC1 signals with the low levels of pERK1/2, pS6K1, and p4E-BP1, and serum re-supply significantly activated both signals with peak activation occurs earlier for mTORC1, and later for the MAPK signals. Interestingly, *SHOC2* knockdown reduced the levels of MAPK reactivation with minimal, if any, effect on the reactivation of the mTORC1 and mTORC2 signals (Fig. 3A and 3B).

We next investigated the effect of SHOC2 on cellular responses to the stimulation of amino acids and glucose. We knocked down *SHOC2* in HepG2, Hep3B, and Huh7 cells, followed by deprivation of amino acids or glucose for 3 h, then resupply of amino acids or glucose for 20 min before harvesting cells for Western blotting. In the absence of amino acids, the mTORC1 signal, as reflected by the levels of pS6K1 was higher in HepG2 and Hep3B cells, but not in Huh7 cells upon *SHOC2* knockdown (Fig. 3C and Fig. S3D). Upon stimulation of amino acids, mTORC1 activation was significantly higher in all three cell lines with *SHOC2* knockdown. Again, amino acid stress had no effect on SHOC2 levels. Interestingly, the mTORC2 or MAPK signals were largely unaffected by amino acids regardless of *SHOC2* knockdown, except that the MAPK signal was even inactivated upon resupply of amino acids in HepG2 cells with *SHOC2* knockdown (Fig. 3C). Similarly, under glucose stimulation, SHOC2 levels were not affected, but *SHOC2* knockdown also triggered great activation of mTORC1 signals with remarkable increase of pS6K1 levels in HepG2 and Hep3B cells. And the effect on mTORC2 or MAPK signals was minor, if any (Fig. 3D). Thus, SHOC2 regulates both MAPK and mTORC1 signals under physiological and stressed conditions, although SHOC2 itself was not subjected to regulations by these stress conditions.

### Hepatocyte-specific *Shoc2* deletion inactivates the Mapk signal but had no effect on liver tumorigenesis induced by DEN-HFD

**Figure 4.**
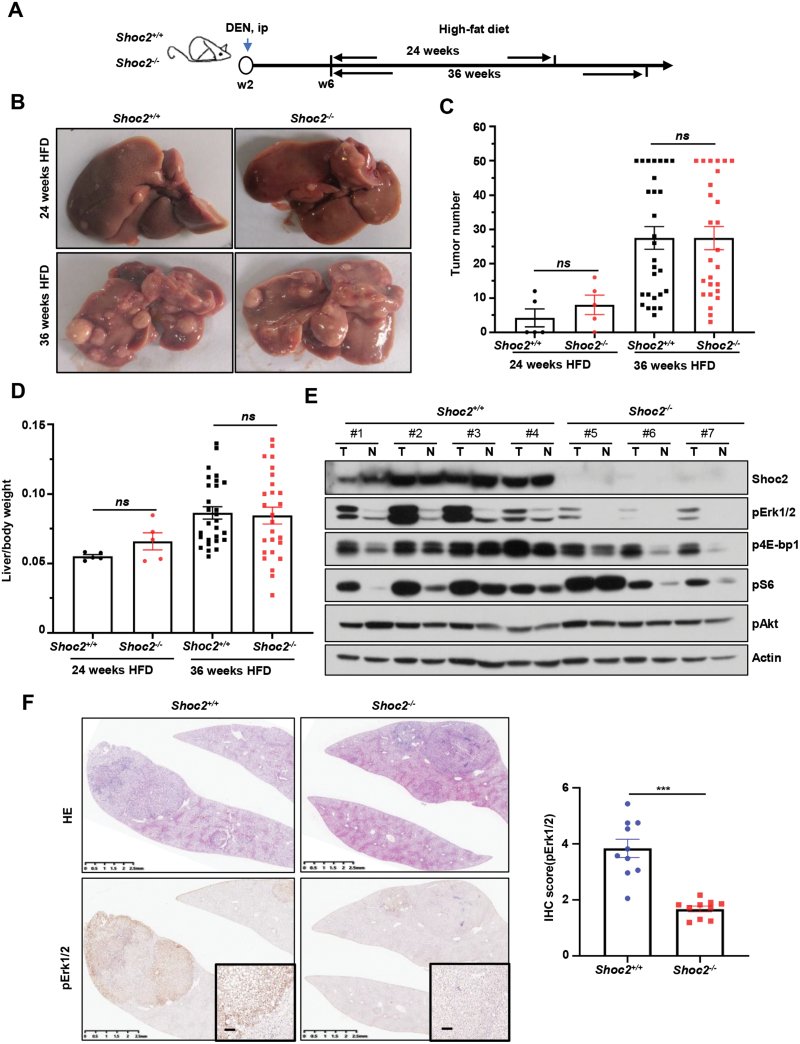
**Hepatocyte-specific *Shoc2* deletion inactivates Mapk signal but had no effect in liver tumorigenesis induced by DEN-HFD.** (A) Experimental design for an i.p. DEN plus HFD-induced liver cancer mouse model. (B) Representative liver images of *Shoc2*^*+/+*^ and *Shoc2*^*−/−*^ mice with the treatment of DEN-HFD. (C, D) Tumor number (C) and the liver/body weight ratio (D) were analyzed in *Shoc2*^*+/+*^ and *Shoc2*^*−/−*^ mice after 24 weeks HFD (*n* = 5 mice/group) and after 36 weeks HFD (*n* = 28 for *Shoc2*^*+/+*^ group, *n* = 26 for *Shoc2*^*−/−*^ group). For all panels, results are expressed as the mean ± SEM of the indicated number of mice per group. (E) Liver tumor tissues and adjacent normal tissues isolated from seven individual mice after 36 weeks HFD with indicated genotypes were milled and lysed, and then subjected to IB with indicated antibodies. (F) Representative images of liver sections from *Shoc2*^*+/+*^ and *Shoc2*^*−/−*^ mice (*n* = 10 mice/group) sacrificed after 36 weeks HFD with H&E staining (top) and immune-histochemical staining (bottom) with indicated antibodies. Scale bars, 100 µm. The staining quantification was analyzed by the semiquantitative immunoreactivity scoring system. DEN: diethylnitrosamine; HFD: high-fat diet; T: liver tumor tissues; N: adjacent normal liver tissues; *ns*: no significance; ***, *P* < 0.001.

We next investigated the role of Shoc2 in *in vivo* liver tumorigenesis induced by either chemical carcinogen (DEN-HFD) or genetic deletion (*Pten* loss), which activates the mTorc signal. We first generated a conditional knockout (KO) mouse with inactivation of Shoc2 in liver, driven by Alb-Cre. The mice were designated as *Shoc2*^*+/+*^ (*Shoc2*^*fl/fl*^) and *Shoc2*^*−/−*^ (*Shoc2*^*fl/fl*^*;Alb-Cre*). In a well-established chemical liver carcinogenesis model (DEN-HFD) [30, 31], the mice were injected via i.p. with hepatic carcinogen diethylnitrosamine (DEN) to initiate the tumorigenesis, followed by a long-term high-fat diet (HFD) feeding regimen for tumor promotion. Mice were euthanized after 24 or 36 weeks HFD, respectively, for HCC development (Fig. 4A). The livers derived from 24-week-HFD *Shoc2*^*+/+*^ and *Shoc2*^*−/−*^ mice were equally enlarged and light-colored with few yet similar numbers of tumors (Fig. 4B and 4C). After 36 weeks HFD, the livers from both genotypes of mice developed a large number and bigger size of tumors, but no significant difference was observed, nor the difference in the ratio of liver-to-body weight (Fig. 4B–D). We harvested liver tumor tissues, along with adjacent normal tissues from *Shoc2*^*+/+*^ and *Shoc2*^*−/−*^ mice after 36-week-HFD and performed Western blotting analysis. We confirmed that the Shoc2 protein was near completely depleted in the livers of *Shoc2*^*−/−*^ mice (Fig. 4E and Fig. S4A). Interestingly, compared to *Shoc2*^*+/+*^ livers, the level of pErk1/2 was remarkably reduced, whereas the levels of pS6 and p4E-bp1 were largely unchanged, but with variations among individual samples, and no changes for pAkt in *Shoc2*^*−/−*^ livers (Fig. 4E and Fig. S4A). We further confirmed this observation, using immunohistochemistry staining of the liver tissues derived from *Shoc2*^*+/+*^ vs. *Shoc2*^*−/−*^ mice (Fig. 4F and Fig. S4B, *n* = 10 for each genotype). Thus, in this chemical carcinogenesis model, *Shoc2* deletion significantly inactivates the Mapk signal, but largely not affecting the mTorc signal. Interestingly and surprisingly, inactivation of the Mapk signal did not affect the progression of liver tumorigenesis in this DEN-HFD model.

### Hepatocyte-specific *Shoc2* deletion activates the mTorc1 signal and promotes liver tumorigenesis induced by *Pten* loss

**Figure 5.**
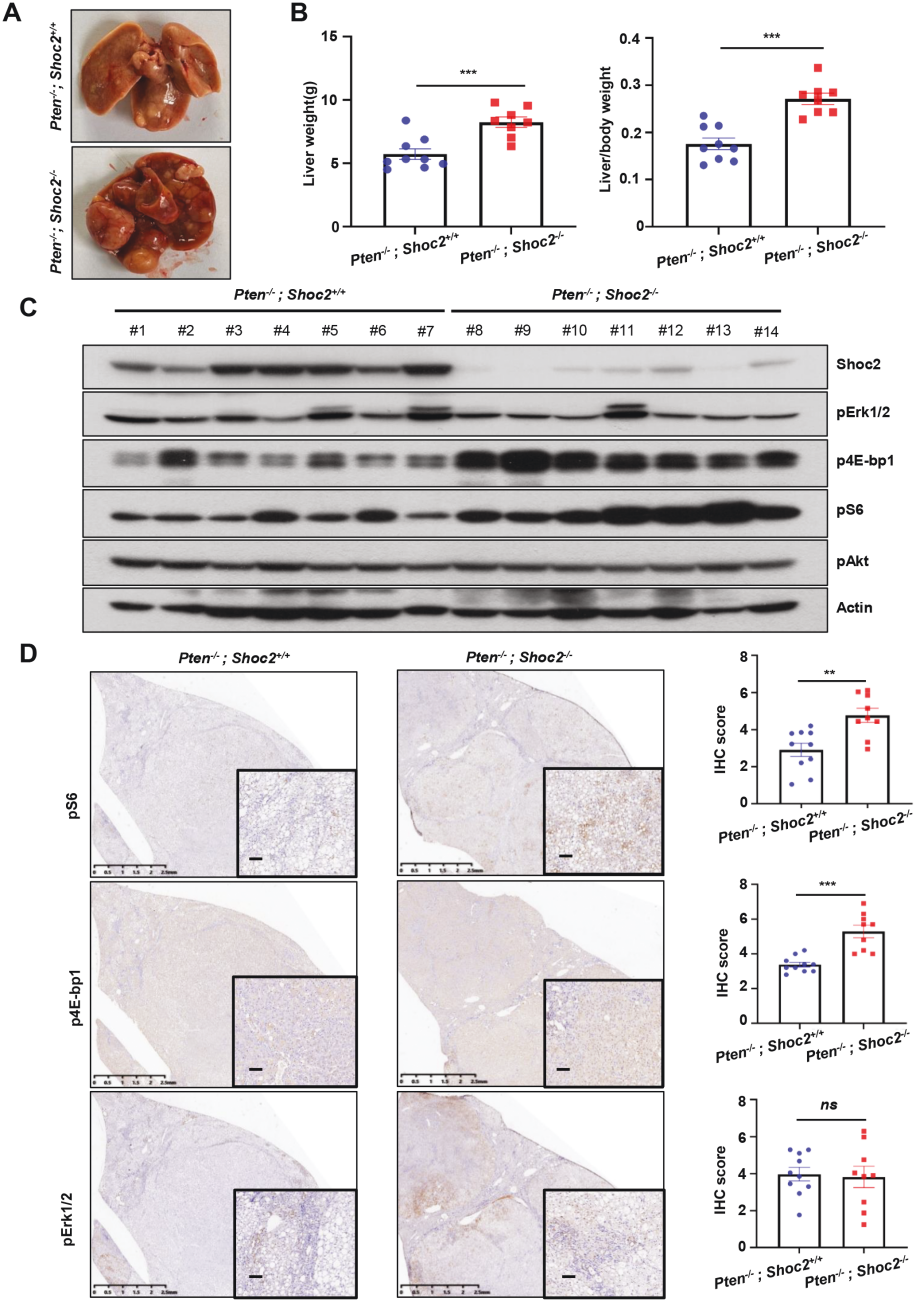
**Hepatocyte-specific *Shoc2* deficiency promotes liver tumorigenesis induced by *Pten* loss.** (A) Representative liver images of *Pten*^*−/−*^*;Shoc2*^*+/+*^ and *Pten*^*−/−*^*;Shoc2*^*−/−*^ mouse at 12 months old. (B) Liver weight and the liver/body weight ratio were analyzed in *Pten*^*−/−*^*;Shoc2*^*+/+*^ and *Pten*^*−/−*^*;Shoc2*^*−/−*^ mouse at 12 months old (*n* = 9 for *Pten*^*−/−*^*;Shoc2*^*+/+*^ group, *n* = 8 for *Pten*^*−/−*^*;Shoc2*^*−/−*^ group). Data are expressed as the mean ± SEM of the indicated number of mice per group. (C) Liver tumor tissues isolated from fourteen individual mice with indicated genotypes were milled and lysed, and then subjected to IB with indicated antibodies. (D) Representative images of liver sections with immune-histochemical staining with indicated antibodies. The liver tissues were isolated from *Pten*^*−/−*^*;Shoc2*^*+/+*^ and *Pten*^*−/−*^*;Shoc2*^*−/−*^ mice (*n* = 10 for *Pten*^*−/−*^*;Shoc2*^*+/+*^ group, *n* = 9 for *Pten*^*−/−*^*;Shoc2*^*−/−*^ group) sacrificed at 12 months old. Scale bars, 100 µm. The staining quantification was analyzed by the semiquantitative immunoreactivity scoring system. ns: no significance; **, *P* < 0.005; ***, *P* < 0.001.

PTEN inactivation is a frequent event in human liver cancer [32], which leads to activation of the mTORC signals [33]. Given the observations that *SHOC2* deletion activates the mTORC1 signal in cell culture models (Fig. 2B) [8], we next investigated whether *Shoc2* deletion would further activate mTorc1 signals, thus promoting liver tumorigenesis in Pten-null liver tumor model. To this end, the *Pten*^*fl/fl*^ mice were crossed with *Shoc2*^*fl/fl*^*;Alb-Cre* mice. After two rounds of mating, we eventually generated two types of compound mice with the genotypes of *Alb-Cre;Pten*^*fl/fl*^*;Shoc2*^*+/+*^ (*Pten*^*−/−*^*;Shoc2*^*+/+*^); and *Alb-Cre;Pten*^*fl/fl*^*;Shoc2*^*fl/fl*^ (*Pten*^*−/−*^*;Shoc2*^*−/−*^). It is worth noting that Pten-loss had no effect on the levels of Shoc2 in the liver tissues, since similar levels were observed between the *Shoc2*^*+/+*^ livers of DEN-HFD model and the *Pten*^*−/−*^*;Shoc2*^*+/+*^ liver tissues (Fig. S5A). Strikingly, the *Pten*^*−/−*^*;Shoc2*^*−/−*^ double null mice developed greater number and larger sized liver tumors than that of *Pten*^*−/−*^*;Shoc2*^*+/+*^ control mice (Fig. 5A) with increased liver weight and increased ratio of liver/body weight (Fig. 5B). Consistently, the H&E staining revealed that the *Pten*^*−/−*^*;Shoc2*^*−/−*^ mice had remarkably elevated tumor burden in the livers (Fig. S5B). The Western blotting showed that Shoc2 was indeed depleted in the liver tumors derived from the *Pten*^*−/−*^*;Shoc2*^*−/−*^ mice, along with significant activation of mTorc1 (p4E-bp1, pS6), but not mTorc2 (pAkt), nor Mapk (pErk1/2) (Fig. 5C). Consistently, immunochemical staining also showed that *Shoc2* deletion significantly activated mTorc1, as reflected by increased staining of p4E-bp1 and pS6, without affecting the staining of pErk1/2 and pAkt (Fig. 5D and Fig. S5C). Taken together, these results demonstrated that *Shoc2* deletion further activates the mTorc1 signal without affecting the Mapk signal and promotes liver tumorigenesis induced by *Pten* loss.

### Correlation between the SHOC2-pERK1/2 and SHOC2-pS6 levels in liver cancer tissues

**Figure 6.**
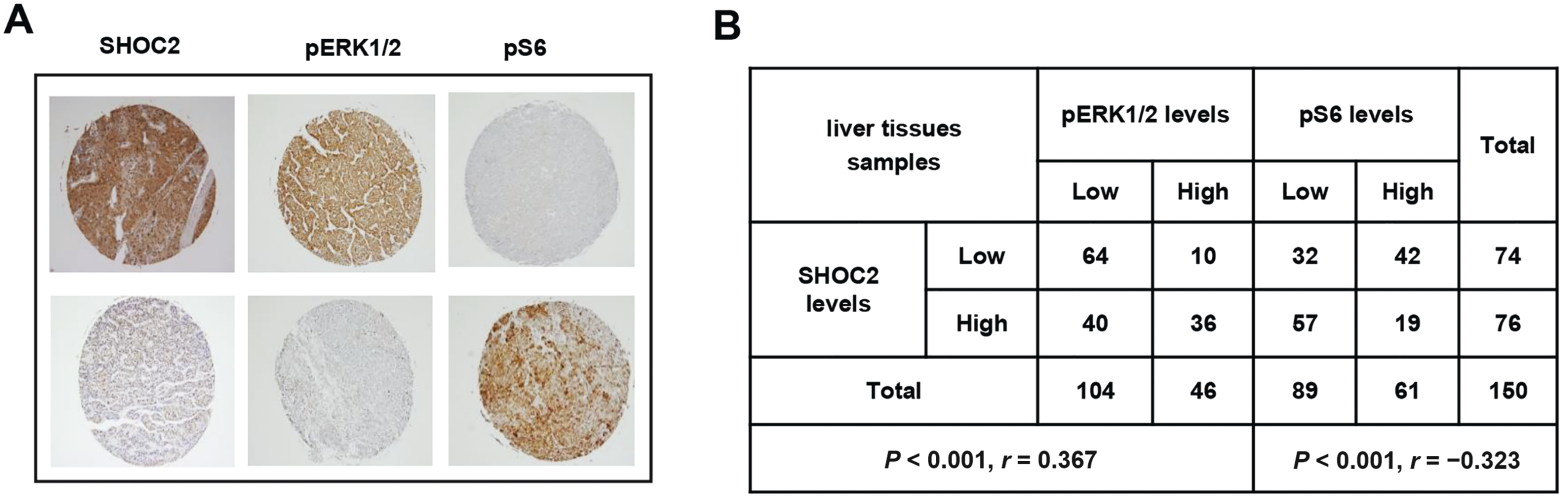
**SHOC2 correlated positively with MAPK, but negatively with the mTORC1 signals.** (A, B) Human liver tumor tissue microarray was stained for the levels of SHOC2, pERK1/2, and pS6 (A) and scored (B) based upon staining intensity. Data were analyzed using SPSS software to obtain correlation coefficient (*P* < 0.001, Pearson’s test).

Finally, we used the IHC staining to measure the levels of SHOC2, pERK1/2 (the MAPK signal), and pS6 (the mTORC1 signal) in liver cancer tissues and the correlations among them in a total of 150 liver tumor tissues. A positive correlation between SHOC2 and pERK1/2 and a negative correlation between SHOC2 and pS6 were found, which is statistically significant (Fig. 6A and 6B). The results highly suggest that SHOC2 is a positive or negative regulator of the MAPK or mTORC1 signals, respectively, in liver cancer.

## Discussion

A majority of SHOC2 studies in defining its role in cancer used human cancer cell lines [11, 14, 34], and only two studies involved the use of genetically modified mouse models, one for lung tumorigenesis [16], and the other for pancreatic tumorigenesis [20], both driven by oncogenic *Kras* mutant (*Kras*^G12D^). In both studies, *Shoc2* acts as a *Kras* cooperative gene, whose deletion inhibited tumorigenesis [16, 20]. With regards to liver cancer, there is only one study, reporting that SHOC2 expression was increased in liver metastases of colon cancer, and *SHOC2* knockdown inhibited the liver metastasis potential of HCT116 cells [12]. In this study, we used multiple *in vitro* liver cancer cell lines, and two *in vivo* liver tumorigenesis models, and concluded that SHOC2 regulates the growth and survival of liver cancer cells *in vitro*, and liver tumorigenesis *in vivo* via modulating the activity of the MAPK and mTORC1 signals. Specifically, the SHOC2-activated MAPK signal plays a dominant role in *in vitro* cell culture models, whereas the Shoc2-inactivated mTorc1 signal plays a major role in the *in vivo Pten*-loss model.

Through an analysis of the TCGA database, comprising 371 cases of liver cancer, we found that *SHOC2* mRNA expression was significantly increased in liver cancer, as compared to normal liver tissue (*P < *0.001). The cell line-based follow-up studies revealed that SHOC2 is required for the growth and clonogenic survival of liver cancer cells, since ectopic expression promoted, whereas siRNA-based knockdown suppressed the growth and survival, indicating that SHOC2 plays an oncogenic role.

The RAS/MAPK pathway plays a fundamental role in the control of many cellular processes, including cell growth and survival [35], and the mTORC1 signal plays a central role in the syntheses of macro-biomolecules such as protein and lipid [36]. Aberrant activations of both pathways are associated with neoplastic transformation and tumorigenesis [37, 38]. The scaffold protein SHOC2 has been identified as a RAS-activator, by either forming a complex with RAS and RAF [4] or by dephosphorylating the inhibitory site of RAF at Ser-259 when SHOC2 is in a complex with the catalytic subunit of protein phosphatase 1 (PP1c) and M-Ras [6, 11]. Our previous study showed that SHOC2 also negatively regulated the mTORC1 signal by competing with mTOR for Raptor binding in lung cancer cells [8]. We reported here that the same observation can be extended to liver cancer cells, given the observations that ectopic SHOC2 expression activated the MAPK, but inactivated the mTORC1 signals, and *vice versa* upon *SHOC2* knockdown. The fact that MAPK activation or inactivation led to growth promotion or suppression, respectively, regardless of the status of mTORC1 activation or inactivation, strongly suggested that the RAS-MAPK signal played a dominant role in SHOC2 regulation of growth and survival in liver cancer cells.

To date, there is no report with regards to whether and how SHOC2 responds to stressed conditions, nor how the MAPK and mTORC1 signals respond to nutrition restriction upon *SHOC2* knockdown. It is well established that the MAPK signal responds rapidly to serum deprivation-resupply [39], whereas the mTORC1 signal responds to deprivation-resupply of amino acids or glucose [40, 41]. Here, we showed that upon *SHOC2* knockdown, the MAPK activation upon serum re-supply to serum-starved liver cancer cells was attenuated, whereas the mTORC1 activation was not significantly affected. On the other hand, SHOC2 knockdown significantly enhanced mTORC1 activation in amino acid or glucose-deprived liver cancer cells upon nutrition resupply, but had a minimal, if any, effect on the MAPK activation. Thus, SHOC2 not only affects the basal levels of MAPK and mTORC1 signals but also regulates its activation upon stressed conditions.

Several studies reported increased levels and activation of the MEK1/2 and ERK1/2 in human liver cancer, as compared to surrounding non-neoplastic liver tissue [42, 43]. In this study, we used a carcinogen-induced mouse liver cancer models (DEN-HFD) to determine the role of Shoc2 in carcinogenesis and found that the pErk1/2 levels were indeed higher in tumor tissues than in adjacent normal tissues regardless of Shoc2 inactivation. On the other hand, *Shoc2* KO significantly reduced the pErk1/2 levels in tumor tissues as well as in adjacent normal tissues. Surprisingly, the inactivation of Mapk signals triggered by *Shoc2* deletion unexpectedly did not translate to reduced tumor burden, suggesting the potential involvement of other pathway genes in driving DEN-initiated liver tumorigenesis. Indeed, the whole exome sequences of mouse liver tumors arising from DEN exposure revealed frequent mutations on *Hras*, *Braf*, and *Egfr*, along with truncation mutation on *Apc* in 21% of tumors [44], suggesting that deregulation of the Wnt/β-Catenin signal could play a major role in DEN-induced liver tumorigenesis. Nevertheless, our data demonstrated that *Shoc2* deletion, although it inactivates the Mapk signal, had no effect on liver carcinogenesis in this chemical carcinogen-induced liver cancer model, suggesting the Mapk signal did not play a major role in the process.

In HCC, about 50% of cases had aberrantly activated PI3K/AKT/mTOR pathway [22, 23], and about 50% of cases harbored PTEN loss or reduced PTEN levels [24]. Thus, The *Alb-Cre;Pten*^*fl/fl*^ mouse strain with liver-specific deletion of *Pten* has been frequently used in the study of liver tumorigenesis [28]. Given that the Pten loss activates the mTORC signal and Shoc2 loss also activates the mTORC1 signal, we tested our working hypothesis that double null for Pten and Shoc2 would accelerate the process of liver tumorigenesis. Indeed, in this Pten-null liver cancer model, *Shoc2* deletion further activated the mTORC1 signal, but not affected the MAPK signal, unlike the cell culture models, in which *SHOC2* deletion significantly inactivated the MAPK signal. More importantly, *Shoc2* deletion significantly promoted liver tumorigenesis. Collectively, the mTorc1 activation is the key determinant for Shoc2 action without involving the Mapk signal in this liver cancer model. Thus, *Shoc2* plays a tissue-specific role, acting as an oncogene-cooperative gene during *Kras*^G12D^-induced tumorigenesis in the lung and pancreas [16, 20], but a tumor suppressor-cooperative gene during Pten-loss-induced tumorigenesis in the liver. In liver cancer tissues, we found that the levels of SHOC2 are positively correlated with MAPK but negatively correlated with mTORC1, further supporting the notion that SHOC2 is a positive regulator of MAPK and a negative regulator of mTORC1 in liver cancer.

Finally, in this study, we made an interesting observation that *SHOC2* knockdown suppressed the growth and survival of liver cancer cells *in vitro*, whereas Shoc2 depletion promoted liver tumorigenesis in *Pten*-loss model. How comes that SHOC2 has an opposite effect *in vitro* vs. *in vivo*? To address this, we knocked down *PTEN* in liver cancer cells and found it activated the mTORC1 signal as expected, but had minor, if any, effect on SHOC2 levels or the RAS-MAPK signals (Fig. S2B and S2C). Furthermore, while double knockdown of *SHOC2* and *PTEN* in cultured liver cancer cells did moderately affect the pERK1/2 and PTEN signals (Fig. 2C and 2D), it was much minor than what was seen in the *in vivo* model in which double KO of *Shoc2* and *Pten* significantly activated the mTorc1, but without affecting pErk1/2 (Fig. 5C). Interestingly, while *PTEN* knockdown promoted, and *SHOC2* knockdown inhibited, respectively, the growth and clonal survival of liver cancer cells (Fig. S3A and S3B), the double knockdown of *PTEN* and *SHOC2* canceled the effect of each other, showing minimal, if any, effect (Fig. S3A–C). The results clearly demonstrated a different consequence between *in vitro* cell growth model in petri dish and *in vivo* tumorigenesis model in mice, although the underlying mechanism remains elusive. Nevertheless, it is not uncommon that a given gene has different functions when being tested *in vitro vs. in vivo* settings.

**Figure 7.**
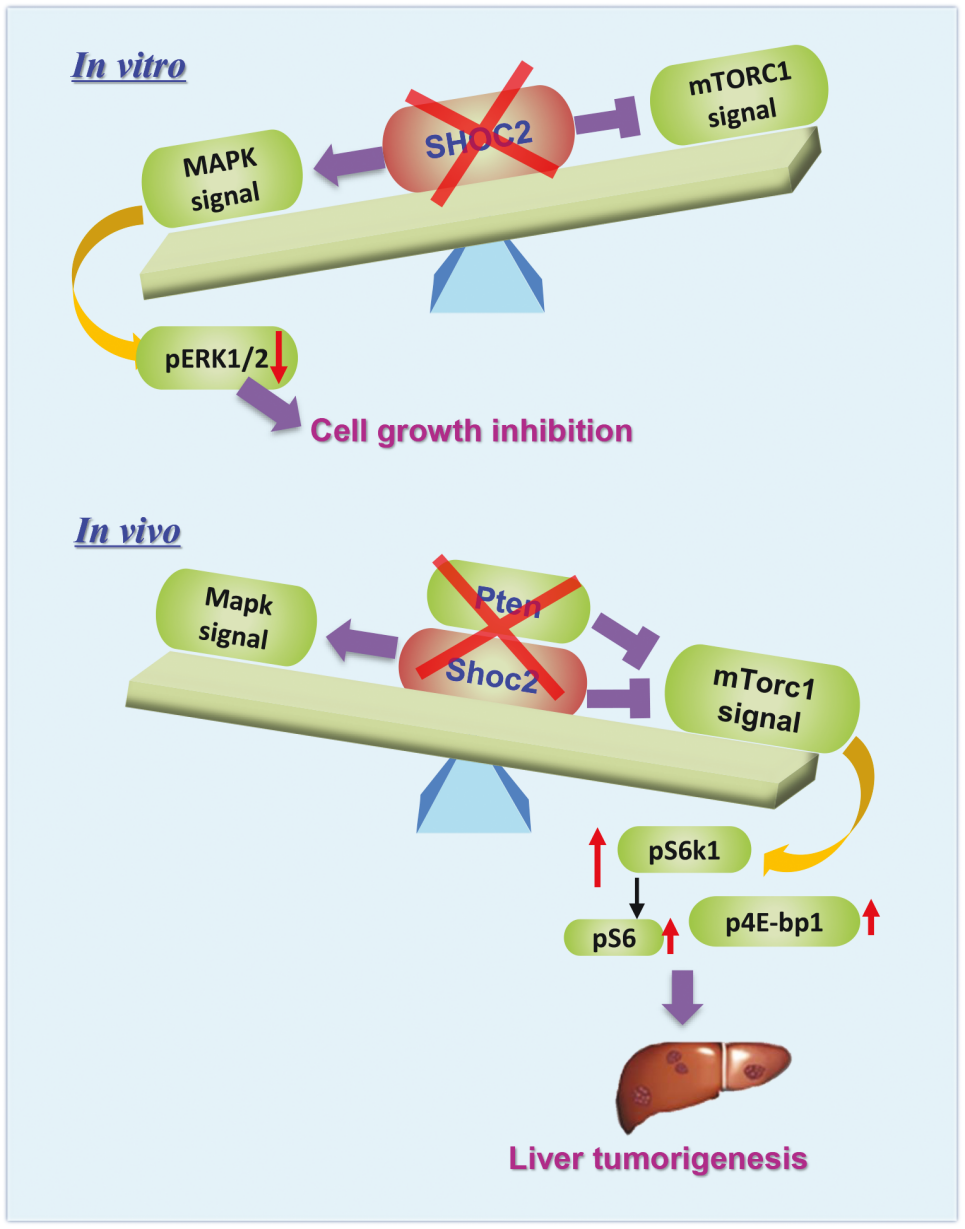
**A working model for differential regulation of SHOC2 on the MAPK and mTORC1 signals in liver cancer cells *in vitro vs.* liver tumors *in vivo*.** In *in vitro* cell culture models, *SHOC2* knockdown inhibits the growth of liver cancer cells, thus acting as an oncogene with greater effect on the MAPK signal than on the mTORC1 signals. In an *in vivo* liver cancer model induced by Pten loss with mTorc1 activation, *Shoc2* deletion promotes liver tumorigenesis, thus acting as a tumor suppressor with greater effect on the mTorc1 signal than on the Mapk signal.

In summary, this is the first study to demonstrate that SHOC2 acts as either oncogene or tumor suppressor by regulating the MAPK and mTORC1 signals in a manner dependent of dominancy of these two signals in liver cancers. Our study fits the following working model. In *in vitro* cell culture models, the activation of MAPK signal is more effective than the inactivation of mTORC1, thereby SHOC2 acts as an oncogene to promote the growth and survival of liver cancer cells. However, in an *in vivo* liver cancer model induced by Pten-loss with mTorc activation, Shoc2 acts as a tumor suppressor, since its deletion further activates the mTorc1 signal to promote liver tumorigenesis (Fig. 7).

## Research limitations

Although we demonstrated here, for the first time, that SHOC2 acts as either an oncogene or a tumor suppressor by differentially regulating the MAPK and mTORC1 signals, using multiple liver cancer cell culture models and two liver tumorigenesis models, our study bears some limitations. Firstly, we did not define the cellular context that determines the role of SHOC2 (*in vitro* vs. *in vivo*), although we did show that its role is dependent of the dominancy between MAPK vs. mTORC1 pathway. Secondly, similarly, the detailed underlying mechanism(s) by which SHOC2 differentially regulates the MAPK and mTORC1 signals in *in vitro* cell culture model vs. *in vivo* tumorigenesis model remains elusive, and awaits for future investigation. Thirdly, it remains to be determined why *Shoc2* deletion did not change the course of liver tumorigenesis, even though the Mapk signal is remarkably inactivated in DEN-HFD liver cancer model, although it is likely and highly suggestive that the Mapk signal is not the driving force in this model.

## Methods

### Transfection of plasmids and siRNAs

The plasmid constructs expressing FLAG-SHOC2 were used as described [8]. The siRNA oligos were obtained from Genepharma. China and the sequences of siRNA oligos are listed in Table S1. Transient transfection of the siRNA oligos or plasmids was performed using Lipofectamine 3000 (Invitrogen), according to the manufacturer’s instructions.

### Immunoblotting (IB)

For IB analysis, cells were lysed in RIPA lysis buffer with protease inhibitor cocktail and phosphatase inhibitors. Supernatants after centrifugation were separated on SDS–PAGE, and then transferred to a nitrocellulose membrane. After blocking with 5% (*w*/*v*) nonfat milk, the membranes were immunoblotted with the indicated antibodies. Antibodies are listed in Table S2.

### Generation of conditional KO mice and PCR-based genotyping

The *Shoc2*^*fl/+*^ mice with floxed exon 3 in *Shoc2* allele were described [20]. *Pten*^*fl/fl*^ mice, which have loxP sites flanking exon 5 of the phosphatase and tensin homolog gene, were obtained from Jackson Laboratory. Alb-Cre mice were obtained from Jackson Laboratory, in which expression of Cre is controlled by the promoter of the hepatocyte-specific gene Albumin. For genotyping, genomic DNA was isolated from the tips of mouse tails and genotyped using the primers listed in Table S3.

All mice were maintained in specific pathogen-free (SPF) conditions with experimental/control groups co-housed. Mice were euthanized via cervical dislocation or carbon dioxide.

### DEN+HFD induced HCC model

Exclusively male mice were used for the HCC model. In DEN-HFD HCC model, 2-week-old male mice were injected intraperitoneally with the hepatic carcinogen DEN (25 mg/kg of mice, Sigma-Aldrich). At the age of 6 weeks, mice were fed an HFD. Mice were sacrificed 24–36 weeks after the HFD feeding to evaluate the development of HCC.

### Immuno-histochemical staining

Mouse liver tissues were harvested and then fixed in 10% formalin and embedded in paraffin. Sections of 5-μm thick were cut for H&E staining and immunohistochemistry. The primary antibodies used in Table S2 and the ABC Vectastain Kit (Vector Laboratories) with secondary antibodies were used for staining, then scanned by a Slide Scanner (Ningbo Konfoong Bio-information Tech).

For quantification, at least five random fields of liver tissues were photographed at 20× magnification and were then analyzed and calculated using the semiquantitative immunoreactivity scoring system. Specifically, stained tissues were classified into four groups according to the staining intensity as follows: negative (0), weak (1), moderate (2), and strong (3). The proportion scores of indicated protein expression depending on the percentage of positive cells were classified as follows: 0% (0), ≤ 10% (1), 11%–50% (2), 51%–80% (3), ≥ 81% (4). The total scores were calculated by multiplying the intensity score by the proportion score [45].

### Tissue microarray and immunohistochemistry

Immunohistochemistry staining of human liver cancer tissue arrays was performed as described above. The stained slides were observed under a microscope (Olympus 1X71), and images were acquired using software DP controller (ver. 3.1.1.267, Olympus). Stained tumor tissues were classified into two groups (low vs. high) according to the staining intensity of each tissue.

### Research ethics

All animal procedures were approved by the Animal Ethics Committee of Zhejiang University. Human liver cancer tissue arrays (LV1003, LV1504, and LV1505) containing 150 tumors were purchased from Alenabio Biotechnology (Chongqing, China), and all samples were from Asian patients. All the methods were carried out in accordance with the institutional protocols and approved by the Ethics Committee of Zhejiang University, Hangzhou, China.

### Statistical analysis

The significance of the data between two experimental groups was determined by Student’s *t*-test, and multiple group comparisons were analyzed by one-way ANOVA. All statistical analyses were two-sided, and different cutoff values, *P *< 0.05 (*), *P* < 0.005 (**), and *P* < 0.001 (***), were considered significant. Pearson’s correlation coefficient was used for parametric variables. The level of significance was *P* < 0.05.

## Supplementary Material

lnae023_suppl_Supplementary_Figures

lnae023_suppl_Supplementary_Materials

## Data Availability

The data supporting the findings of this study are available within the article and its supplementary materials.
