# Supplementary material for: SHOC2 plays an oncogenic or tumor-suppressive role by differentially targeting the MAPK and mTORC1 signals in liver cancer
Source: Life Med. 2024 May 23;3(3):lnae023. doi: 10.1093/lifemedi/lnae023 (PMC11749279; doi:10.1093/lifemedi/lnae023)
Supplement: lnae023_suppl_Supplementary_Figures [file lnae023_suppl_Supplementary_Figures.pptx]

## Slide 1
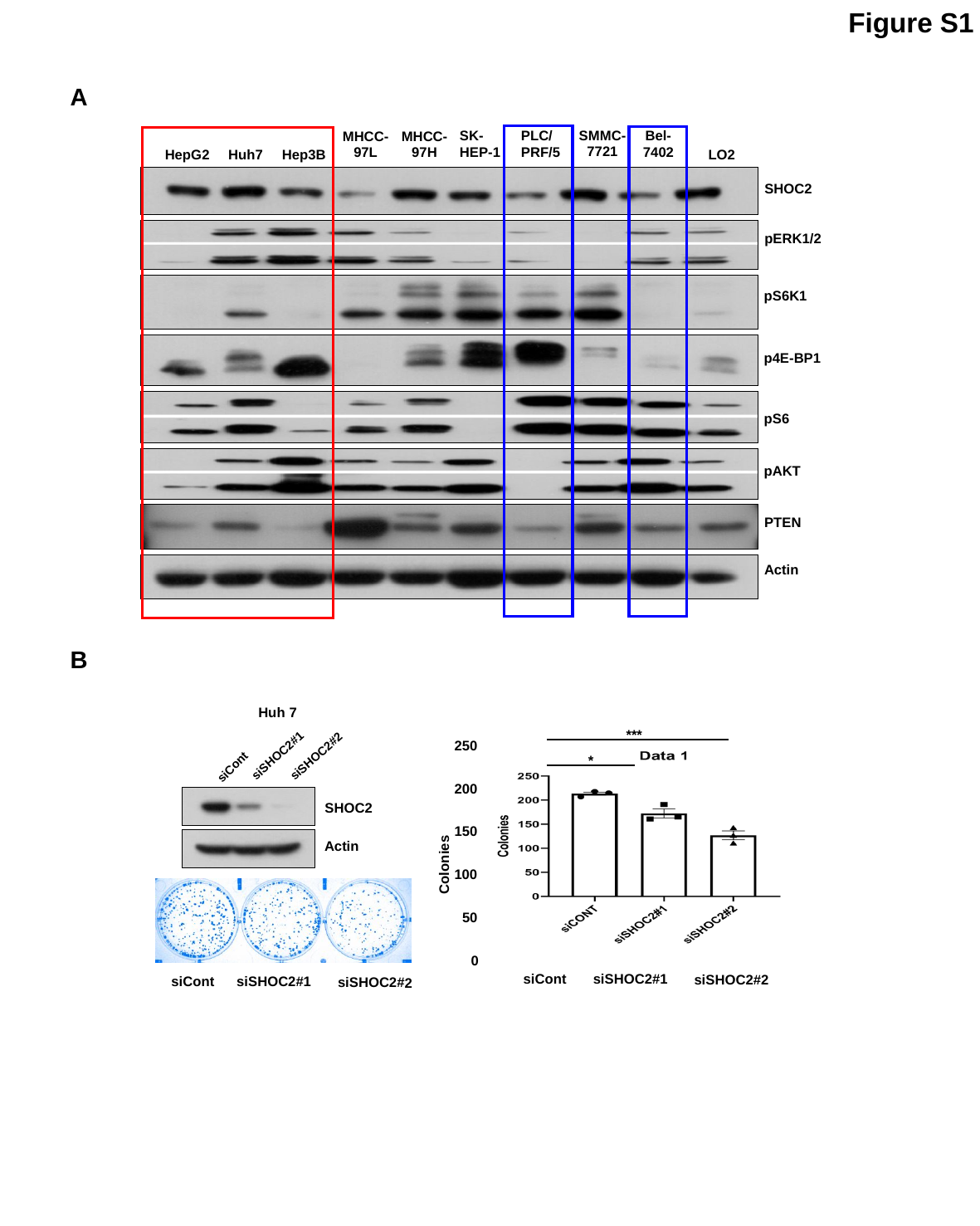

Figure S1
A
SMMC-
7721
SK-
HEP-1
PLC/
PRF/5
Bel-
7402
MHCC-
97L
MHCC-
97H
LO2
HepG2
Huh7
Hep3B
SHOC2
pERK1/2
pS6K1
p4E-BP1
pS6
pAKT
PTEN
Actin
B
Huh 7
siSHOC2#1
siSHOC2#2
siCont
SHOC2
Actin
siCont
siSHOC2#1
siSHOC2#2
***
250
*
200
150
Colonies
100
50
0
siCont
siSHOC2#1
siSHOC2#2

## Slide 2
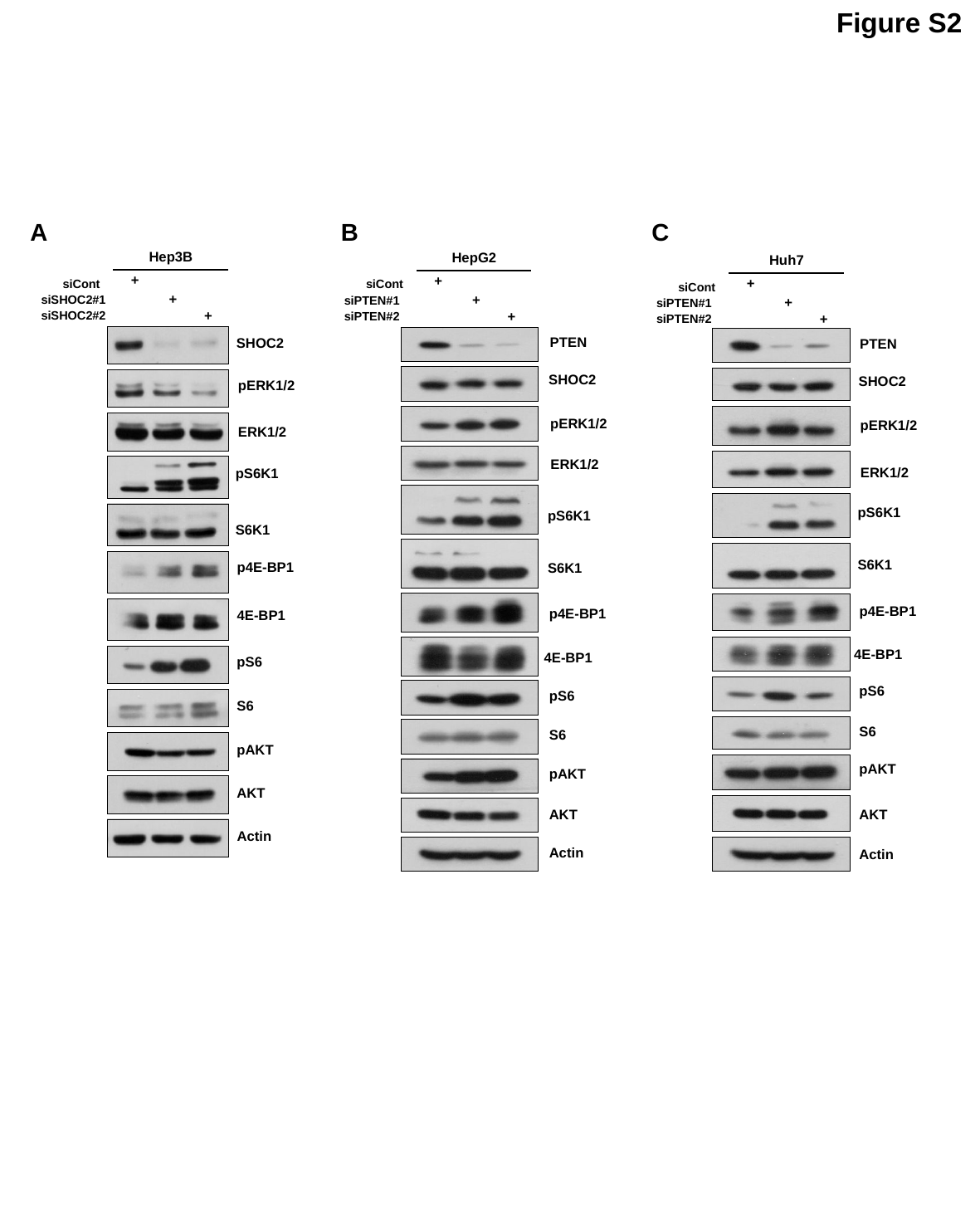

Figure S2
A
B
C
Hep3B
+
+
+
siCont
siSHOC2#1
siSHOC2#2
SHOC2
pERK1/2
ERK1/2
pS6K1
S6K1
p4E-BP1
4E-BP1
pS6
S6
pAKT
AKT
Actin
HepG2
+
+
+
siCont
siPTEN#1
siPTEN#2
PTEN
SHOC2
pERK1/2
ERK1/2
pS6K1
S6K1
p4E-BP1
4E-BP1
pS6
S6
pAKT
AKT
Actin
Huh7
+
+
+
siCont
siPTEN#1
siPTEN#2
PTEN
SHOC2
pERK1/2
ERK1/2
pS6K1
S6K1
p4E-BP1
4E-BP1
pS6
S6
pAKT
AKT
Actin

## Slide 3
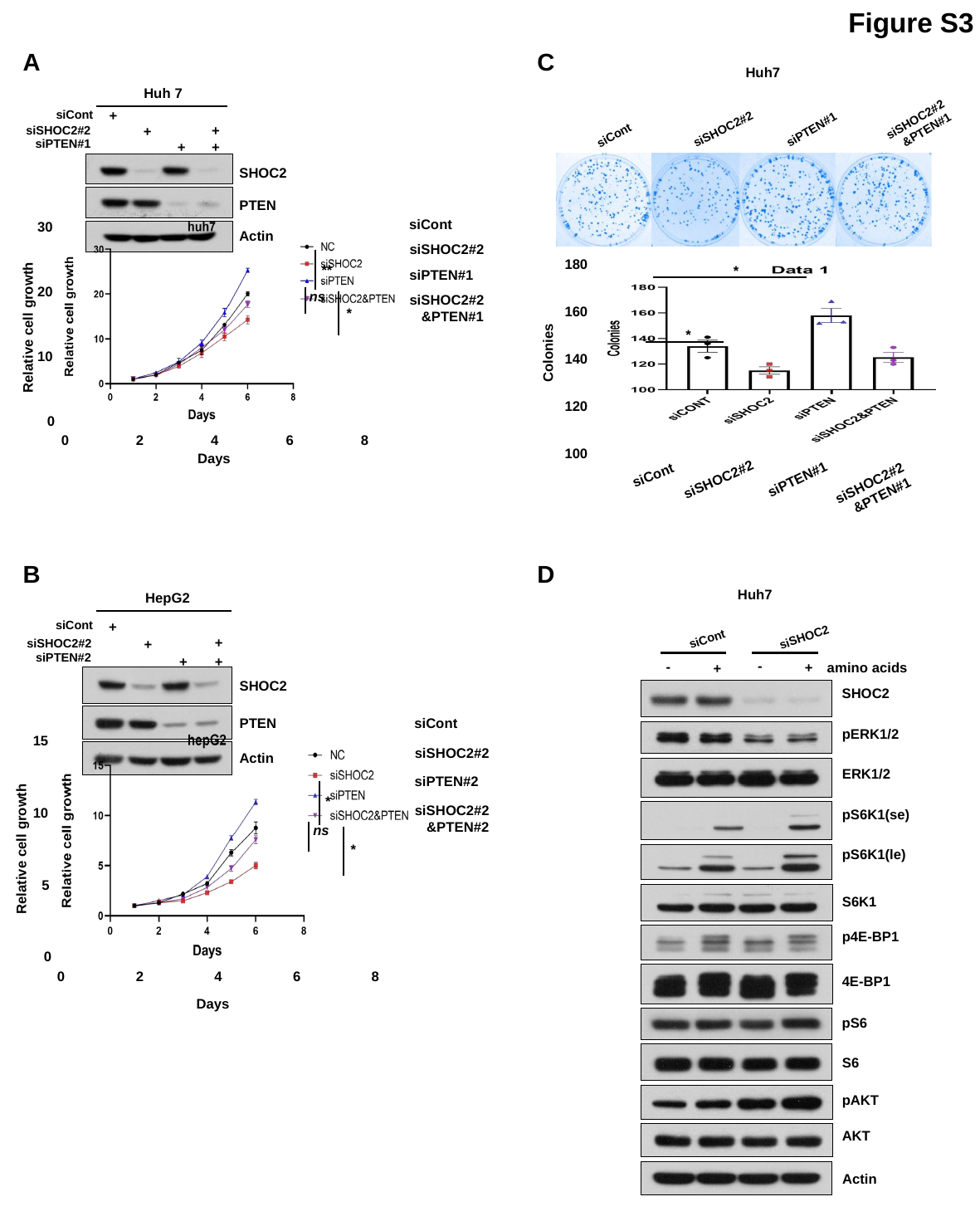

Figure S3
A
C
Huh7
Huh 7
+
siCont
+
+
siSHOC2#2
siPTEN#1
+
+
SHOC2
PTEN
Actin
siSHOC2#2
 &PTEN#1
siSHOC2#2
siPTEN#1
siCont
siCont
30
20
10
0
siSHOC2#2
siPTEN#1
siSHOC2#2
 &PTEN#1
Relative cell growth
0
2
4
6
8
Days
**
ns
*
180
160
140
120
100
Colonies
siCont
siPTEN#1
siSHOC2#2
siSHOC2#2
 &PTEN#1
*
*
B
D
Huh7
siCont
siSHOC2
-
-
amino acids
+
+
SHOC2
pERK1/2
ERK1/2
pS6K1(se)
pS6K1(le)
S6K1
p4E-BP1
4E-BP1
pS6
S6
pAKT
AKT
Actin
HepG2
siCont
+
+
+
siSHOC2#2
siPTEN#2
+
+
SHOC2
PTEN
Actin
siCont
15
10
5
0
siSHOC2#2
siPTEN#2
siSHOC2#2
 &PTEN#2
Relative cell growth
0
2
4
6
8
Days
*
ns
*

## Slide 4
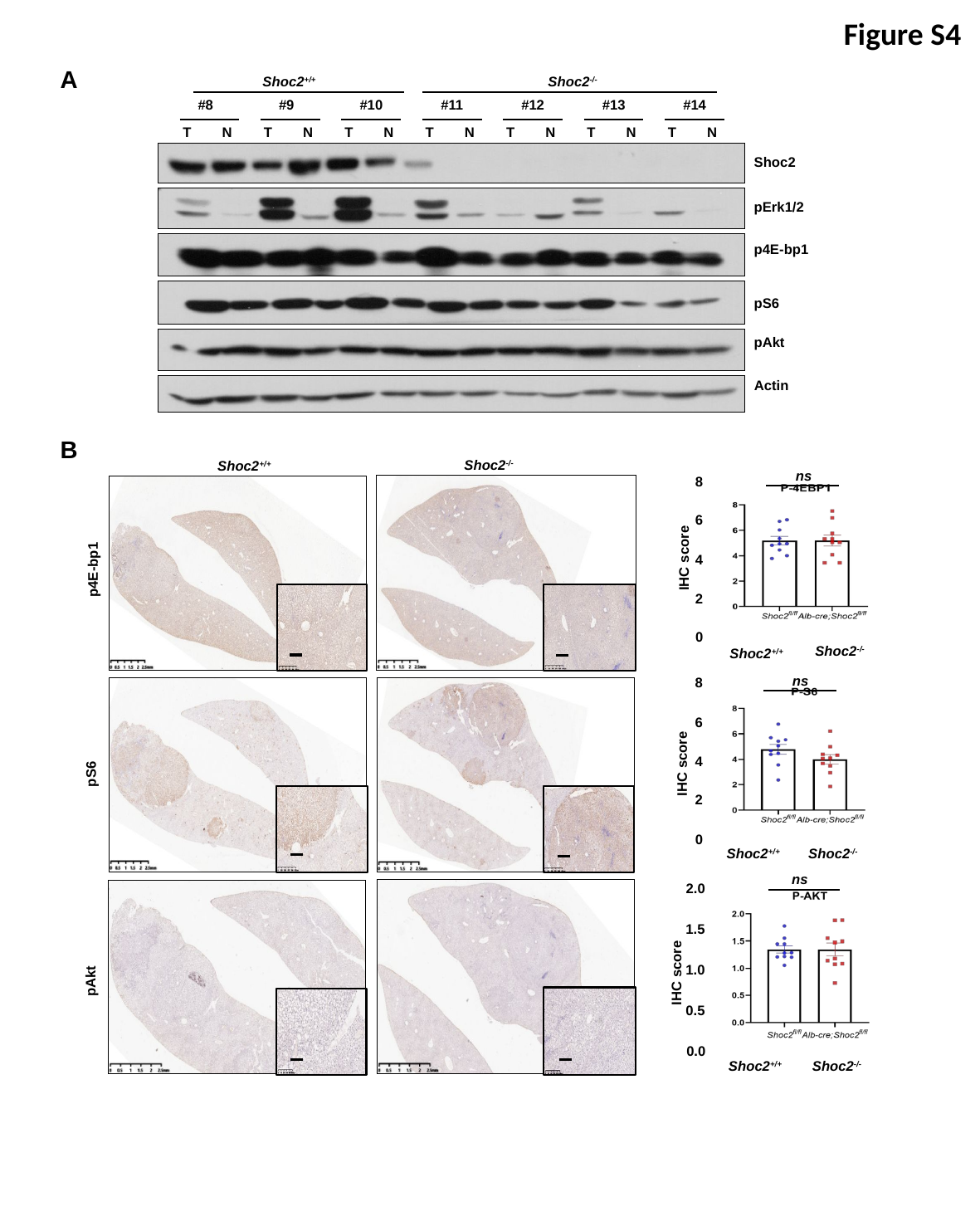

Figure S4
A
Shoc2+/+
Shoc2-/-
#8
T
N
#9
T
N
#10
T
N
#11
T
N
#12
T
N
#13
T
N
#14
T
N
Shoc2
pErk1/2
p4E-bp1
pS6
pAkt
Actin
B
Shoc2-/-
Shoc2+/+
p4E-bp1
pS6
pAkt
ns
8
6
4
2
0
IHC score
Shoc2-/-
Shoc2+/+
ns
8
6
4
2
0
IHC score
Shoc2+/+
Shoc2-/-
ns
2.0
1.5
1.0
0.5
0.0
IHC score
Shoc2-/-
Shoc2+/+

## Slide 5
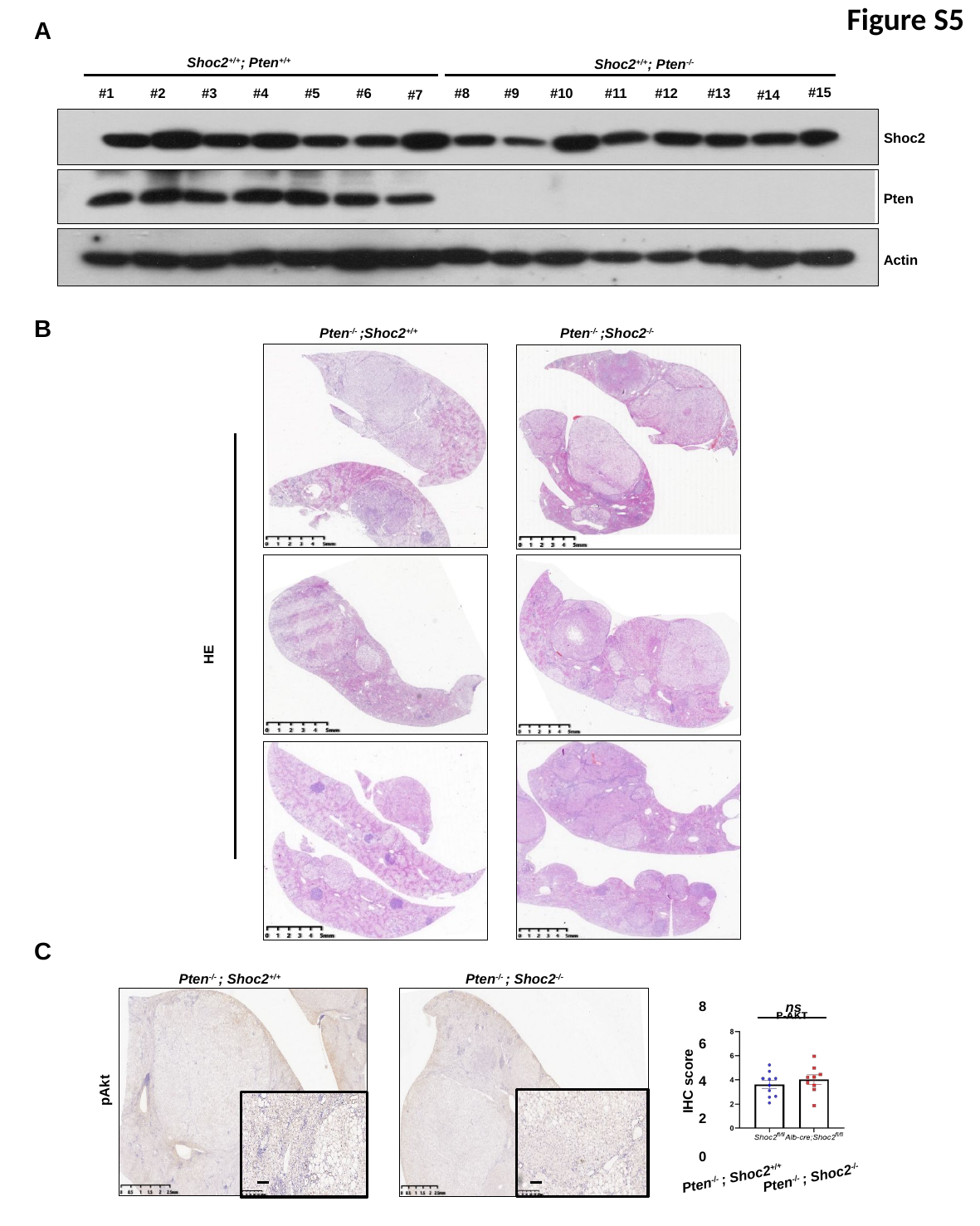

Figure S5
A
Shoc2+/+; Pten+/+
#1
#2
#3
#4
#5
#6
#8
#9
#10
#11
#12
#13
#7
#14
Shoc2
Pten
Actin
Shoc2+/+; Pten-/-
#15
B
Pten-/- ;Shoc2+/+
Pten-/- ;Shoc2-/-
HE
C
Pten-/- ; Shoc2+/+
pAkt
Pten-/- ; Shoc2-/-
8
6
4
2
0
ns
IHC score
Pten-/- ; Shoc2-/-
Pten-/- ; Shoc2+/+
