# Supplementary material for: SHOC2 plays an oncogenic or tumor-suppressive role by differentially targeting the MAPK and mTORC1 signals in liver cancer
Source: Life Med. 2024 May 23;3(3):lnae023. doi: 10.1093/lifemedi/lnae023 (PMC11749279; doi:10.1093/lifemedi/lnae023)
Supplement: lnae023_suppl_Supplementary_Materials [file lnae023_suppl_Supplementary_Materials.docx]

**Supplemental materials**

**SHOC2 plays an oncogenic or tumor suppressive role**

**by differentially targeting the MAPK and mTORC1 signals in liver cancer**

Xiahong You^1,2,^, Longyu Dou^1,2,^, Mingjia Tan^3^, Xiufang Xiong^1,2^, and Yi Sun^1, 2, 4, 5#^

^1^Cancer Institute (Key Laboratory of Cancer Prevention and Intervention, China National Ministry of Education) of the Second Affiliated Hospital and Institute of Translational Medicine, Zhejiang University School of Medicine, Hangzhou 310029, China

^2^Cancer Center of Zhejiang University, Hangzhou 310029, China

^3^ Department of Radiation Oncology, University of Michigan, Ann Arbor, MI 48109, USA

^4^Zhejiang Provincial Clinical Research Center for CANCER, Hangzhou 310029, China

^5^Research Center for Life Science and Human Health, Binjiang Institute of Zhejiang University, Hangzhou 310053, China

^#^ Correspondence: yisun@zju.edu.cn (Y.S.)

**Running title:** SHOC2 regulation of the RAS and mTORC1 signals

**Key Words:** SHOC2, RAS, mTORC1, PTEN, liver cancer.

**Materials and Methods:**

**The Cancer Genome Atlas Data Analysis**

The data files on liver hepatocellular carcinoma patients were downloaded from the Cancer Genome Atlas (TCGA) dataset and processed according to the operational processes of the public data provider. In total, we analyzed 50 normal liver tissues and 371 liver tumor samples.

**Cell culture**

The HepG2, Hep3B, PLC/PRF/5, Bel7402, Huh7, MHCC-97L, MHCC-97H, SK-HEP-1, SMMC7721 liver cancer cell lines, and the normal immortalized liver LO2 cells were maintained in high glucose Dulbecco’s MEM supplemented with 10% FBS and 1% penicillin/streptomycin solution. All cells were cultured in a 37°C humidified incubator with 5% CO_2_.

**Cell proliferation and clonogenic survival assay**

Cell proliferation was measured using Cell Counting Kit-8 (CCK-8, MedChem Express) according to the manufacturer’s instructions. For clonogenic survival assay, 500-1000 cells were seeded into 35 mm dishes in triplicate and grown for 14 days. The colonies were fixed, stained and counted.

**Supplemental Figure legends:**

**Figure S1. The basic level of SHOC2 in human liver cell lines.**

(A) HepG2, Hep3B, PLC/PRF/5, Bel-7402, Huh7, MHCC-97L, MHCC-97H, SK-HEP-1, SMMC7721 liver cancer cells, and normal liver LO2 cells were lysed in lysis buffer with protease inhibitor cocktail and phosphatase inhibitors, followed by IB with indicated antibodies. (B) Huh7 cells were transfected with siRNA targeting *SHOC2* or control siRNA (siCont) for 24 h, followed by clonogenic survival assay. Representative pictures were taken (left) and colonies were counted and are shown as mean ± SEM (*n* = 3) (right). *, *p* < 0.05; ***, *p* < 0.001.

**Figure S2. SHOC2 positively regulates MAPK, but negatively regulates mTORC1 signals**.

(A) Hep3B cells were transfected with siRNA targeting *SHOC2* or siCont for 48 h, followed by IB with indicated antibodies. (B and C) HepG2 (B) and Huh7 (C) cells were transfected with siRNA targeting *PTEN* or siCont for 48 h, followed by IB with indicated antibodies.

**Figure S3. SHOC2 and PTEN regulation of growth and survival, and SHOC2 regulation of the MAPK and mTORC1 signals under stress conditions.**

(A and B) Huh7 (A) and HepG2 (B) cells were transfected with indicated siRNA or control siRNA (siCont) for 24 h. Cells were then seeded in 96-well plates in triplicate and subjected to a CCK-8 cell proliferation assay or IB (inset). (C) Huh7 cells were transfected with indicated siRNA or control siRNA (siCont) for 24 h, followed by clonogenic survival assay. Representative pictures were taken (top) and colonies were counted and are shown as mean ± SEM (*n* = 3) (bottom). (D) Huh7 cells were transfected with siRNA targeting SHOC2 or scrambled control siRNA (siCont) for 48 h, followed by amino acids starvation for 3 h, then amino acids re-supply for 20 minutes and harvested for IB. se: short exposure; le: long exposure. *, *p* < 0.05; **, *p* < 0.005; ns: no significance.

**Figure S4. Inactivation of *Shoc2* inhibits the Mapk, but not the mTorc1 signaling in liver cancer induced by DEN-HFD.**

(A) Liver tumor tissues and adjacent normal tissues isolated from seven individual mice with indicated genotypes were milled and lysed, and then subjected to IB with indicated antibodies. (B) Histological analyses of liver sections from *Shoc2^+/+^* and *Shoc2^−/−^* mice (*n* = 10 mice/group) sacrificed after 36 weeks of HFD. The liver tissues were isolated from mice, fixed, sectioned and and subjected to immunohistochemical staining with indicated antibodies. The staining quantification was analyzed by the semiquantitative immunoreactivity scoring system as described in the materials and methods section. T: tumor tissues; N: normal liver tissues; *ns*: no significance. Scale bars, 100 µm.

**Figure S5. Inactivation of *Shoc2* promotes liver tumorigenesis in *Pten*-loss model.**

(A) *Pten* deletion has no effect on Shoc2 levels. Liver tumor tissues isolated from fifteen individual mice at age of 12 months with indicated genotypes were homogenized and lysed, and then subjected to IB with indicated antibodies. (B and C) *Shoc2* deletion promotes tumorigenesis in *Pten*-loss model, but had no effect on the mTorc2 signal. Histological analyses of liver sections from *Pten^−/−^;Shoc2^+/+^* and *Pten^−/−^;Shoc2^−/−^* mice (*n* = 10 for *Pten^−/−^;Shoc2^+/+^* group, *n* = 9 for *Pten^−/−^;Shoc2^−/−^* group) sacrificed at 12 months old. The liver tissues were isolated from mice, fixed, sectioned and subjected to H&E staining (B) and immune-histochemical staining (C) with indicated antibodies. The staining quantification was analyzed by the semi-quantitative immunoreactivity scoring system as described in the materials and methods section. ns: no significance. Scale bars, 100 µm.

**Table S1. The sequences of siRNA oligos used in this study**

| **Name** | **siRNA targeting sequence** |
| --- | --- |
| siSHOC2#1 | 5'-AAGCTGCGGATGCTTGATT-3' |
| siSHOC2#2 | 5'-TACCTTCGCTTTAATCGTATA-3' |
| siPTEN#1 | 5'-CTAGAACTTATCAAACCCTTT-3' |
| siPTEN#2 | 5'-GACGGGAAGACAAGUUCAUTT-3' |

**Table S2. A list of primary antibodies used in this study**

| **Antibody** | **Sources (Catalogue Number)** |
| --- | --- |
| **Western blotting** |  |
| SHOC2 Rabbit mAb | Abcam (ab106430) |
| pAKT S473 Rabbit mAb | Cell signaling Technology (4060) |
| AKT Rabbit mAb | Cell signaling Technology (4691S) |
| pS6K1 T389 Rabbit mAb | Cell signaling Technology (9234) |
| S6K1 Rabbit mAb | Cell signaling Technology (9202S) |
| pS6 Rabbit mAb | Cell signaling Technology (4858S) |
| S6 Rabbit mAb | Cell signaling Technology (2217) |
| p4E-BP1 Rabbit mAb | Cell signaling Technology (2885) |
| 4E-BP1 Rabbit mAb | Cell signaling Technology (9452) |
| pERK1/2 Rabbit mAb | Cell signaling Technology (4376) |
| ERK1/2 Mouse mAb | Cell signaling Technology (9107) |
| PTEN Rabbit mAb | Cell signaling Technology (9559) |
| Actin Mouse mAb | Sigma (A5441) |
| **Immunohistochemistry** |  |
| pAkt Rabbit mAb | Cell signaling Technology (4060) |
| pS6 Rabbit mAb | Cell signaling Technology (4858S) |
| p4E-BP1 Rabbit mAb | Cell signaling Technology (2885) |
| pERK1/2 Rabbit mAb | Cell signaling Technology (4376) |

**Table S3. The sequences of primers for PCR-based genotyping**

| **Name** | **Forward primer sequence** | **Reverse primer sequence** |
| --- | --- | --- |
| *Shoc2* | 5'-CCTCTAAACCTAACCCTCTTTGAGC-3' | 5'-GCACAGTGTCAGAACCTGGAATCTC-3' |
| *Pten* | 5'-CAAGCACTCTGCGAACTGAG -3' | 5'-AAGTTTTTGAAGGCAAGATGC-3' |
| Alb-Cre | 5'-GCATAACCAGTGAAACAGCATTGCTG-3' | 5'-GGACATGTTCAGGGATCGCCAGGCG-3' |
